# Structural Identification and Conversion Analysis of Malonyl Isoflavonoid Glycosides in Astragali Radix by HPLC Coupled with ESI-Q TOF/MS

**DOI:** 10.3390/molecules24213929

**Published:** 2019-10-31

**Authors:** Yunfeng Zheng, Weiping Duan, Jie Sun, Chenguang Zhao, Qizhen Cheng, Cunyu Li, Guoping Peng

**Affiliations:** 1 School of Pharmacy, Nanjing University of Chinese Medicine, Nanjing 210023, China; weipingduan0805@126.com (W.D.); sunjiexueyong@163.com (J.S.); zcg0925@126.com (C.Z.); Qizhencheng@163.com (Q.C.); licunyuok@163.com (C.L.); 2 Jiangsu Collaborative Innovation Center of Chinese Medicinal Resources Industrialization, Nanjing 210023, China

**Keywords:** malonyl isoflavonoid glycosides, structural identification, conversion analysis, Astragali Radix, ESI/Q-TOF/MS

## Abstract

In this study, four malonyl isoflavonoid glycosides (MIGs), a type of isoflavonoid with poor structural stability, were efficiently isolated and purified from Astragali Radix by a medium pressure ODS C_18_ column chromatography. The structures of the four compounds were determined on the basis of NMR and literature analysis. Their major diagnostic fragment ions and fragmentation pathways were proposed in ESI/Q-TOF/MS positive mode. Using a target precursor ions scan, a total of 26 isoflavonoid compounds, including eleven malonyl isoflavonoid glycosides coupled with eight related isoflavonoid glycosides and seven aglycones were characterized from the methanolic extract of Astragali Radix. To clarify the relationship of MIGs and the ratio of transformation in Astragali Radix under different extraction conditions, two MIGs (calycosin-7-*O*-glycoside-6″-*O*-malonate and formononetin-7-*O*-glycoside-6″-*O*-malonate) coupled with related glycosides (calycosin-7-*O*-glycoside and formononetin-7-*O*-glycoside) and aglycones (calycosin and formononetin) were detected by a comprehensive HPLC-UV method. Results showed that MIGs could convert into related glycosides under elevated temperature conditions, which was further confirmed by the conversion experiment of MIGs reference compounds. Moreover, the total contents of MIGs and related glycosides displayed no obvious change during the long-duration extraction. These findings indicated that the quality of Astragali Radix could be evaluated efficiently and accurately by using the total content of MIGs and related glycosides as the quality index.

## 1. Introduction

Astragali Radix, named “huangqi” in Traditional Chinese Medicine, is the dried root of *Astragalus membranaceus* (Fisch.) Bge. or *A. membranaceus var. mongholicus* (Bge.) Hsiao (family Leguminosae) [1]. As a commonly used natural herb, Astragali Radix has been listed in the Pharmacopoeia of many countries, including the Pharmacopoeia of the People’s Republic of China, the United States Pharmacopoeia, and Japanese Pharmacopoeia. It exhibits many biological functions, such as antioxidative [2,3], anticarcinogenic [4], immunomodulation [5], and anti-inflammatory activities [6], so it is frequently used to treat diabetes and related complications [7]. 

Chemical investigations have shown that isoflavonoids, triterpene saponins, and polysaccharides are the main active components in Astragali Radix [8,9]. Among them, isoflavonoids, as natural compounds that promote human health, were established to be important active components [10]. When the quality of Astragali Radix and its products are evaluated, the inherent isoflavonoids were generally selected as “marker compounds” since their chromophores are suitable for UV detection [11]. However, the criteria and quality assessment methods of isoflavonoids for Astragali Radix are obviously different among the pharmacopoeias and publications. For example, in the Pharmacopoeia of the People’s Republic of China, the active constituent calycosin 7-*O*-β-D-glycoside is used for the quality control of Astragali Radix, while in the United States Pharmacopoeia, the total content of four isoflavonoids (calycosin 7-*O*-β-D-glycoside, ononin, calycosin and formononetin) should be no less than 0.03%. Of note, Astragali Radix contains a group of malonyl isoflavonoid glycosides (MIGs) [12,13,14]. Although MIGs are in relatively high abundance in the raw materials of Astragali Radix, they are seldom investigated for quality evaluation owing to their poor structural stability and unavailability of reference compounds. 

To determine the profiles of MIGs in Astragali Radix, the first step of this study was to isolate MIGs using a medium pressure ODS C_18_ column chromatography (MPLC). Their structures were then elucidated by MS and NMR analysis. Coupling HPLC with Quadrupole Time of Flight Mass Spectrometers (Q-TOF/MS), which shows great advantages in providing accurate mass measurements, resolving power, and high sensitivity [15,16,17], allows unequivocal structural identification of low levels of ingredients and non-target compounds in complicated analysis of herbs. Therefore, a comprehensive HPLC coupled with Q-TOF/MS was developed to characterize MIGs, related glycosides and aglycones in Astragali Radix. Finally, in order to clarify the stability and conversion of MIGs, six major isoflavonoids, including two MIGs, two related glycosides, and two aglycones, were analyzed and compared under different extraction techniques and times.

## 2. Results and Discussion 

### 2.1. Compound Separation and Structural Identification

**Figure 1:**
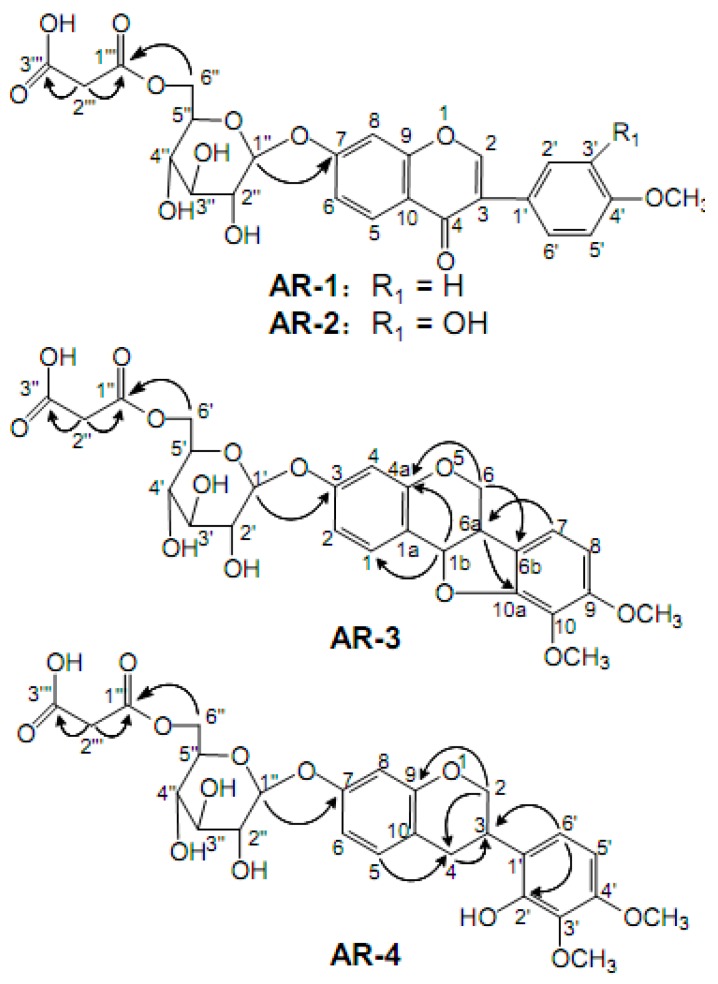
Chemical structures and key HMBC correlations of four malonyl isoflavonoid glycosides isolated from Astragali Radix.

Medium pressure ODS C_18_ column chromatography (MPLC) has been widely used in isolation and purification of natural compounds, organic synthetic molecules, and biological macromolecules [18]. The MPLC column used was packed with 45–60 μm ODS silica gelto provide a short separation time and high separation efficiency of isoflavonoids [19]. The method was primarily based on the selection of experimental conditions in sample adjustment and elution steps. With simple pretreatment procedures and a short separation time, a phytochemical investigation of Astragali Radix by MPLC led to the isolation of our MIG compounds. The structures and the key HMBC correlation of compounds **AR-1** to **AR-4** are illustrated in Figure 1. The ^13^C-NMR and ^1^H- NMR data of the four compounds are shown in Table 1.

**AR-1** was obtained as a light yellow powder and its molecular formula was determined as C_25_H_24_O_12_ based on the HR-ESI-MS at *m/z* 517.1341 [M + H]^+^ (calcd 517.1321). Based on the ^1^H-NMR spectra, three aromatic proton signals at *δ*_H_ 8.08 (1H, d, *J* = 8.8 Hz), *δ*_H_ 7.15 (1H, dd, *J* = 8.8 Hz, *J* = 2.2 Hz), and *δ*_H_ 7.23 (1H, d, *J* = 2.2 Hz) represented the structure containing the AMX spin-coupling. The signal at *δ*_H_ 8.40 (1H, s) was an obvious isoflavonoid signal at the H-2 position. In addition, the AA′BB′ spin-coupling system, with signals at *δ*_H_ 7.53 (2H, d, *J* = 8.0 Hz) and *δ*_H_ 7.00 (2H, d, *J* = 8.0 Hz), was attributed to the four protons of the B-ring. These characteristic features implied that **A** was a typical isoflavonoid compound. The carbohydrate chain of **A** consisted of one monosaccharide residue ascribing the signals of an anomeric carbon at *δ*_C_ 99.7, which was deduced from the ^13^C NMR spectra. This carbohydrate chain was correlated with the corresponding signals of anomeric proton at *δ*_H_ 5.15 (1H, d, *J* = 7.5 Hz) in the HSQC spectrum. The coupling constant of the anomeric protons indicated that the glycosidic bond had a *β*-configuration. The positive ESI-TOF/MS of **AR-1** produced a protonated ion at *m*/*z* 517.1341 [M + H]^+^, as well as the secondary dissociation mass fragment ion at *m*/*z* 269.0801 [M-C_6_H_10_O_5_-C_3_H_2_O_3_ + H]^+^, indicating that the carbohydrate chain consisted of one glucoside and malonyl, which was confirmed by the signals of two carboxyl carbons at 168.0 and 166.9 ppm, one anomeric carbon at 99.7 ppm, four tertiary carbons at 69.7–76.0 ppm, and two secondary carbons at 41.5 and 64.1 ppm. The determination of the linkage sites was obtained from the HMBC correlations between *δ*_H_ 5.15 (H-1′) and *δ*_C_ 161.2 (C-7), as well as *δ*_H_ 4.12 (H-6′) and *δ*_C_ 166.9 (C-1′). Comparing the data with the literature [20,21], the compound **AR-1** was identified as formononetin-7-*O*-β-D-glycoside-6″-*O*-malonate.

The compound **AR-2** was obtained as a light yellow powder. It produced a protonated ion at 533.1267 [M + H]^+^ (calcd 533.1290) in the HR-ESI-MS, corresponding to the molecular formula of C_25_H_24_O_13_, thus it had only one oxygen atom more than **AR-1**. When comparing the ^1^H NMR and ^13^C NMR spectral data of **AR-2** with the data from **AR-1**, the spectral data were very similar; further spectroscopic analysis revealed that the only difference was in the region of the B-ring. In the ^1^H-NMR spectrum, an ABX spin coupling system in **AR-2** rather than AA′ BB′ in **AR-1** were observed, indicating the presence of a hydroxyl moiety at C-3′ in the B-ring of aglycone. Thus, the compound was clarified as calycosin-7-*O*-β-D-glycoside-6″-*O*-malonate, which was further verified by comparison with the literatures [20,21].

**AR-3**, a white powder, generated a [M + NH_4_]^+^ ion at *m*/*z* 566.1839 and a [M + H]^+^ at *m*/*z* 549.1581, which is in agreement with the molecular formula of C_26_H_28_O_13._ The fragment ion peaks at *m*/*z* 301.1063 [M-C_6_H_10_O_5_-C_3_H_2_O_3_ + H]^+^, as well as two secondary carbons at *δ*_C_ 64.3 and 43.1, four tertiary carbons at *δ*_C_ 69.8–76.3, two carboxyl carbons at *δ*_C_ 166.8 and 167.9, and one anomeric carbons *δ*_C_ 100.1 suggested the presence of malonyl glucoside moiety. The ^13^C NMR spectrum of compound **AR-3** exhibited 17 carbon resonances assigned to the aglycone moiety consisting of two OCH_3_, one CH_2_, seven CH (five aromatic, two aliphatic), and seven quaternary carbons (all aliphatic).

The comparative analysis between the ^13^C-NMR spectrum of **AR-3** and **AR-2** showed that the main difference was in the C-ring region. The signals of two methines (*δ*_C_ 39.5, *δ*_C_ 78.2) and one methylene (*δ*_C_ 65.8) in **AR-3,** rather than two unsaturated quaternary carbons (*δ*_C_ 123.6, *δ*_C_ 174.7) and one unsaturated methylene (*δ*_C_ 153.5) in **AR-2** were observed, indicating that **AR-3** was a type of pterocarpan compound. The correlations in the HMBC spectrum from *δ*_H_ 4.28, 3.65 (H-6) to *δ*_C_ 156.1 (C-4a) and 121.6 (C-6a), from *δ*_H_ 6.99 (H-7) to *δ*_C_ 39.5 (C-6a), and from *δ*_H_ 3.65 (H-6a) to *δ*_C_ 150.9 (C-10a) confirmed the compound type of **AR-3**. From these spectroscopic data and the literature information [12,22], compound **AR-3** was identified to be astraperocarpan-3-*O*-β-D-glycoside-6′-*O*-malonate.

**AR-4** was obtained as a white powderwith a molecular formula of C_26_H_30_O_13_ as determined by HR-ESI-MS at *m/z* 568.1991 [M + NH_4_]^+^ ion and *m*/*z* 551.1736 [M + H]^+^ (calculated 365.1759), two more hydrogens than **AR-3**. The ^13^C-NMR spectrum of **AR-4** was similar to the spectrum of **AR-3**, and detail spectroscopic analysis revealed the signal of one methylene at *δ*_C_ 29.7 (C-4) in **AR-4** rather than one oxygenated methane (*δ*_C_ 78.2) as in **AR-3**. These data suggested that **AR-4** was derived from **AR-3** with the ring-opening of its furan ring. This structure was further confirmed by the HSQC correlations between *δ*_H_ 2.92, 2.81(2H, m, H-4) and *δ*_C_ 29.7 (C-4), together with HMBC correlations from *δ*_H_ 4.19 (H-2α) and *δ*_H_ 7.01 (H-5) to *δ*_C_ 29.7 (C-4). Based on the above discussion and the literature [22], **AR-4** was identified as astraisoflavanglycoside-6″-*O*-malonate.

### 2.2. LC-QTOF/MS Analysis of the Extract of Astragali Radix

**Figure 2:**
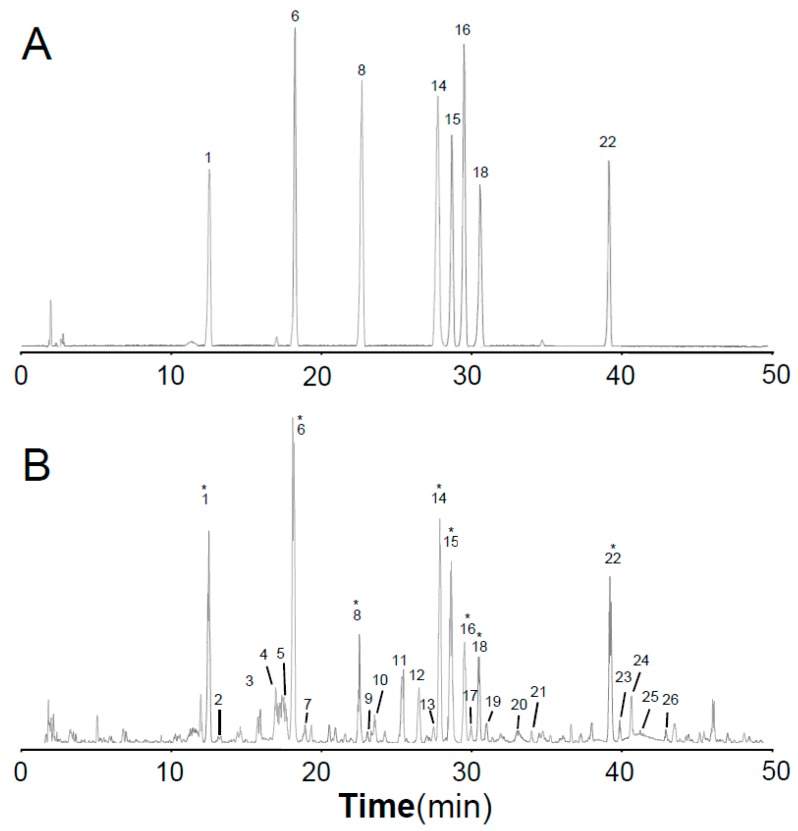
LC-QTOF/MS total ion current (TIC) profile of eight isoflavonoid references (**A**) and the extract of Astragali Radix (**B**) operated in positive mode. * These compounds were identified by their corresponding references.

**Figure 3:**
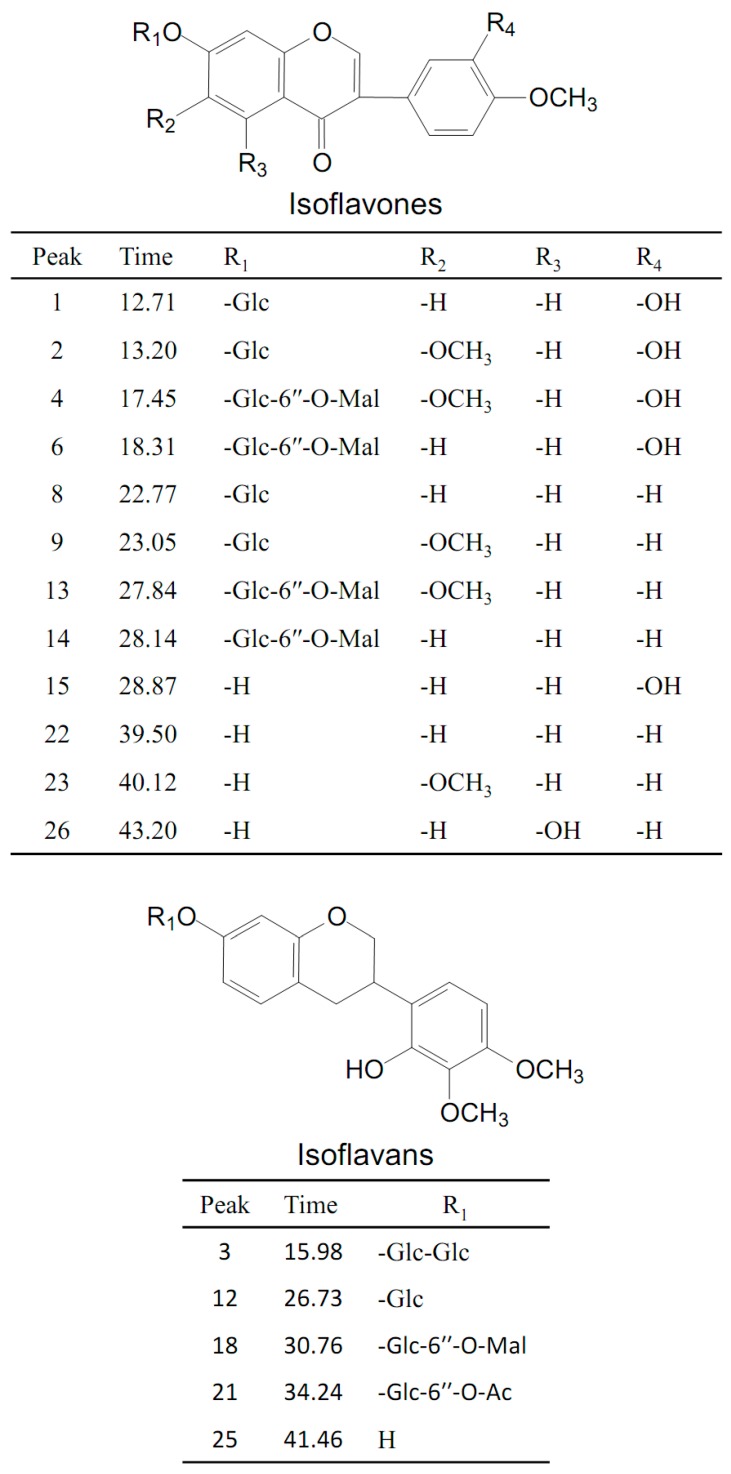

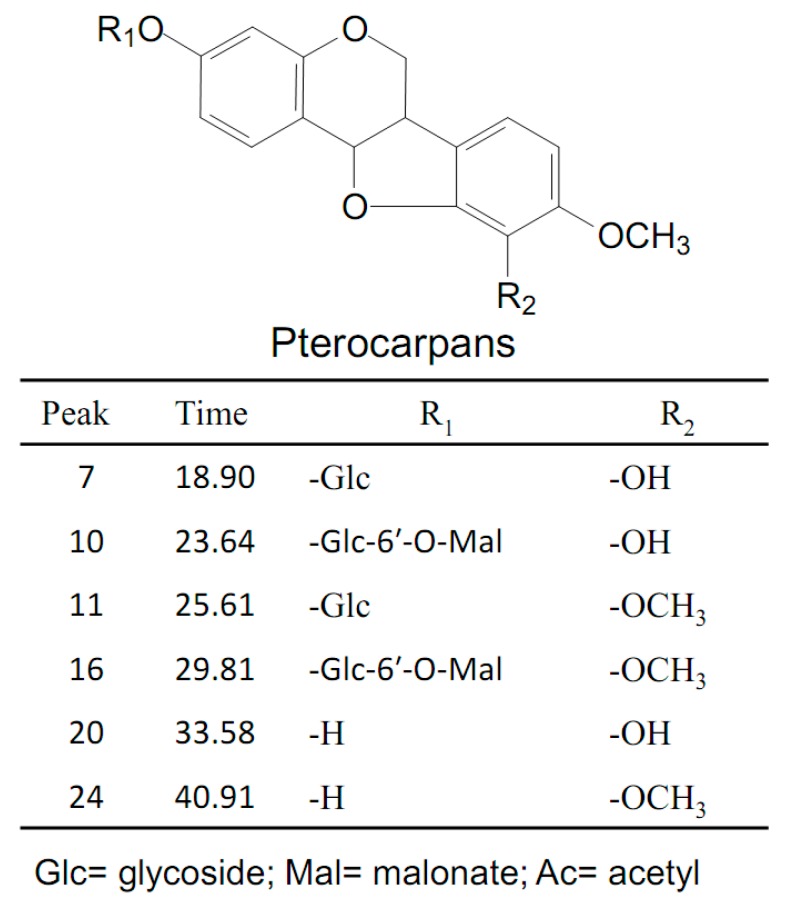
Chemical structures of the isoflavonoid compounds identified in Astragali Radix.

The total ion chromatograms (TICs) of stock solution with eight reference standards and the extract of Astragali Radix in positive mode are shown in Figure 2. The eight references were divided into three groups based on the structural characteristics: isoflavones including calycosin-7-*O*-glycoside (**1**), calycosin-7-*O*-glycoside-6″-*O*-malonate (**6**), formononetin-7-*O*-glycoside (**8**), formononetin-7-*O*-glycoside-6″-*O*-malonate (**14**), calycosin (**15**), and formononetin (**22**); isoflavans consisting of astraisoflavanglycoside-6″-*O*-malonate (**18**); and pterocarpans comprising astraperocarpan-3-*O*-glycoside-6′-*O*-malonate (**16**). A total of 26 isoflavonoids were identified, and their structures are presented in Figure 3. As shown in Table 2, each authentic compound had a significant and distinctive [M + H]^+^ or [M + NH_4_]^+^ ion, MS fragment ions, and quite different retention times. Therefore, they could be unambiguously identified in the extract of Astragali Radix. Other isoflavonoids could be characterized by the developed strategy [23]: First, the molecular formulas of unknown compounds could be obtained by accurate mass measurement and screened in chemical databases such as SciFinder Scholar. Second, typical dissociations of the skeleton were helpful for predicting chemical groups and substituent numbers, and diagnostic ions from [M + H]^+^ were useful for determining substituent moieties. Finally, the number of candidates was sharply reduced after their substructures were identified by the structurally summarized diagnostic ions. If a compound had been reported in plants, especially in the genus *Astragalus*, it was the most proper candidate.

#### 2.2.1. Identification of Malonyl Isoflavone Glycosides and Related Aglycones

**Figure 4:**
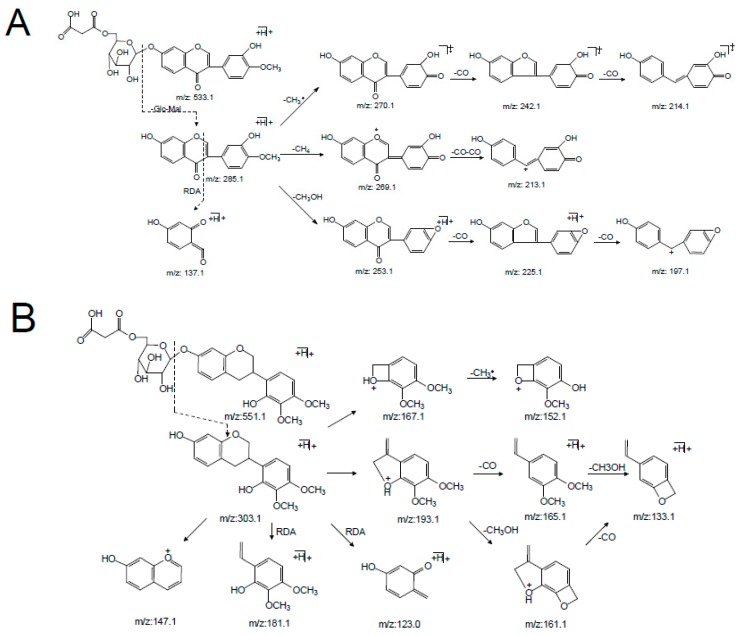

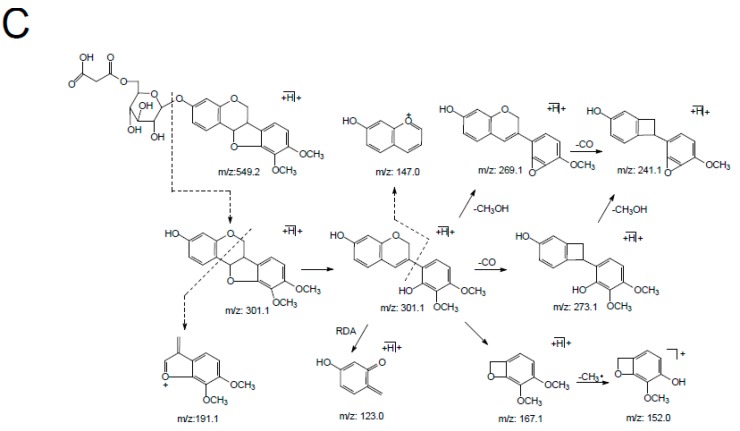
The main MS^n^ fragmentation pathway for three malonyl isoflavonoid glycosides from Astragali Radix. (**A**) Calycosin-7-*O*-Glc-6″-*O*-Mal. (**B**) Astraisoflavanglycoside-6″-*O*-Mal. (**C**) Astraperocarpan-3-*O*-Glc-6′-*O*-Mal.

Peaks **6** and **14** were unambiguously identified as calycosin-7-*O*-glycoside-6″-*O*-malonate and formononetin-7-*O*-glycoside-6″-*O*-malonate, respectively, which were confirmed by comparing their *t_R_* values and mass spectra with those of the references. In the positive mode (supplementary materials Figure S1), the [M + H]^+^ ion of calycosin-7-*O*-glycoside-6″-*O*-malonate (**6**) at *m/z* 533.1260 (C_25_H_24_O_13_) first lost the 248 Da free radical at the carbohydrate chain, yielding the aglycone ion at *m/z* 285.0740 [M + H-C_6_H_10_O_5_-C_3_H_2_O_3_]^+^ (named[Aglycone + H]^+^) as its predominant fragmentation, which further produced ions at *m/z* 137.1 corresponding to the RDA cleavage of a C-ring. From the isoflavone aglycone ion at *m/z* 285.0740 [Aglycone + H]^+^, the MS^2^ spectra revealed that the loss of 15 Da (a methyl radical), 16 Da (CH_4_), or 32 Da (CH_3_OH) led to the formation of the fragmentation ions at *m/z* 270.1 [Aglycone + H-CH_3_]^+^, *m/z* 269.1 [Aglycone + H-CH_4_]^+^, or *m/z* 253.1 [Aglycone + H-CH_3_OH]^+^, respectively, representing the characteristic fragmentation pathway of 4′-methoxylisoflavones [24]. In addition, the base peak at *m/z* 285.0740 [Aglycone + H]^+^ also showed a concurrent loss of CH_3_· and CO or 2CO, concurrent loss of CH_4_ and 2CO, and concurrent loss of CH_3_OH and CO or 2CO, forming the significant ions of [Aglycone + H-CH_3_-CO]^+^, [Aglycone + H-CH_3_-2CO]^+^, [Aglycone + H-CH_4_-2CO]^+^, [Aglycone + H-CH_3_OH-CO]^+^, and [Aglycone + H-CH_3_OH-2CO]^+^, respectively. These product ions allowed us to propose a fragmentation pathway for the [M + H]^+^ ion of calycosin-7-*O*-glycoside-6″-*O*-malonate (Figure 4A and Table 1).

Peak **4** showed an intense ion at *m/z* 563.1323 [M + H]^+^ (C_26_H_26_O_14_), which produced an aglycone ion at *m/z* 315.0867 by losing 248 Da (162 + 86 Da), corresponding to a malonyl glucose residue. In the MS^2^ spectrum, peak **4** was different from peak **6** by an increase of 30 Da (CH_2_O) by the base ion at *m/z* 315.1 [Aglycone + H]^+^ and characteristic RDA fragmentation ion at *m/z* 167.1, indicating that the difference between peak **4** and **6** was the substituted group at the A-ring. With the comparison with the literature [21], peak **4** was tentatively identified as odoratin-7-*O*-glycoside-6″-*O*-malonate. Peak **13** showed an intense ion at *m/z* 547.1421 [M + H]^+^, corresponding to the molecular formula C_26_H_26_O_13_. In LC-MS^2^spectrum, the characteristic fragment ion at *m/z* 167.1 produced in the RDA cleavage was the same as that of **4**, indicating that the difference between peak **13** and **4** was the substituted group at the B-ring. In addition, other fragmented ions at *m/z* 284.1, 283.1, 267.1, and 239.1 visible the *m/z* 299.1 indicated one less hydroxyl in the aglycone structure than peak **4**. According to the literature [24], peak **13** could be considered as afrormosin-7-*O*-glycoside-6″-*O*-malonate.

Peaks **1**, **2**, **8,** and **9** each indicated aglycone ions at *m/z* 285.1, 315.1, 269.1, and 299.1, originating from the loss of 162 Da, respectively, which indicated they were isoflavone glycosides consisting of one glucose residue. Among them, peaks **1** and **2** were unambiguously identified as calycosin-7-*O*-glycoside and formononetin-7-*O*-glycoside, respectively, by comparing the peaks with reference compounds. Peaks **8** and **9** could be tentatively characterized asodoratin-7-*O*-glycoside and 6, 4′-dimethoxyisoflavone-7-*O*-Glc by the MS^2^ spectrum where the product ions were identical with those of peaks **4** and **13**, respectively. Moreover, peaks **15**, **22,** and **23** were considered aglycone of peaks **1**, **8**, and **9**, respectively, because their MS^2^ characteristic ions were identical with the MS^3^ spectrum of peaks **1**, **8**, and **9**.

#### 2.2.2. Analysis of Malonyl Isoflavan Glycosides and Related Aglycones 

The retention time of astraisoflavanglycoside-6″-*O*-malonate (peak **18**) was determined to be 30.76 min by comparison with the reference compound. The [M + NH_4_]^+^ and [M + H]^+^ of peak **18** were at *m/z* 551.1738 and 568.2004, respectively. In the positive ion mode of the LC-MS^n^ spectrum (supplementary materials Figure S2), the product ions at *m/z* 515.1 and 497.1 were from [M + H]^+^ by the successive eliminations of 2 × 18 Da (2H_2_O) and 3 × 18 Da (3H_2_O). In addition, the typical loss of 86 Da (*m/z* 497.1→411.1) and 248 Da (*m/z* 551.1→303.1) were observed in the MS^2^ spectrum, corresponding to the malonyl and malonyl glucose residue. The other product ions at *m/z* 193.1, 181.1, 167.1, 165.1, 161.1, 152.1, 147.1, 133.1, and 123.0 were produced from aglycone ion *m/z* 303.1 due to C-ring RDA cleavage, as well as arrangement at and successive loss of CH_3,_ CO, and CH_3_OH (Figure 4B). These characteristic ions were identical with the MS^2^ spectra of peak **25,** leading to the identification of peak **25** as isomucronulatol.

Peaks **3**, **12** and **21** all showed a distinctive aglycone ion at *m/z* 303.1, resulting from the loss of carbohydrate units (two glucoses (2 × 162 Da), one glucose (162 Da), and an acetylglucose (42 + 162 Da), respectively). According to their identical characteristic aglycone product ions with isomucronulatol and typical neutral losses, peaks **3** and **12** were tentatively deduced as isomucronulatol-7-*O*-glycoside-glycoside and astraisoflavanglycoside, respectively, while peak **21** was tentatively characterized as isomucronulatol-7-*O*-glycoside-6″-*O*-acetyl.

#### 2.2.3. Characterization of Malonyl Pterocarpan Glycosides and Related Aglycones

Peak **16** was unambiguously identified as astraperocarpan-3-*O*-glycoside-6′-*O*-malonate, which was confirmed by the comparison of its *t_R_* and mass spectra with the reference standard. The intense [M + NH_4_]^+^ and weak ion [M + H]^+^ of peak **16** was at *m/z* 549.1584 and 566.1846, respectively. In the LC-MS^n^ spectrum (supplementary materials Figure S3), successive losses of several H_2_O and malonyl molecules from the *m/z* 549.1 [M + H]^+^ produced *m/z* 513.1 [M + H-2H_2_O]^+^, 495.1 [M + H-3H_2_O]^+^, and 409.1 [M + H-3H_2_O-C_3_H_2_O_3_]^+^. The distinctive aglycone fragment at *m/z* 301.1 was formed due to the neutral loss of 248 Da (malonyl glucose moiety) from [M + H]^+^. The product ions at *m/z* 273.1, 269.1, and 241.1 arose from the aglycone ion by concurrent loss of CH_3_OH (32Da) and CO (28Da) fragments; the ion at m/z 123.0 was produced from the RDA cleavage of the aglycone ion. In addition, the other product ions were at *m/z* 191.1, 167.1, 152.1 and 147.0 due to the losses of the B-ring and C-ring arrangement (Figure 4C). This result indicated that peak **16** (pterocarpans) exhibited similar MS/MS fragmentation features to that of peak **18** (isoflavans). The main difference between peaks **16** and **18** was that pterocarpans yielded the typical ions by the concurrent loss of CH_3_OH and CO from [Aglycone + H]^+^ ion, where asisoflavans did not show the same characteristic losses.

Peak **11** produced precursor ions at *m/z* 463.1579 [M + H]^+^ and *m/z* 480.1841 [M + NH_4_]^+^, indicating the molecular formula of C_23_H_26_O_10_. The MS^2^ spectra yielded the aglycone ion at *m/z* 301.1 by the loss of 162 Da (glucose), and their characteristic product ions at *m/z* 273.1, 269.1, 241.1, 191.1, 167.1, 152.0, 147.0 and 123.0, identical to MS^2^ spectra of peak **16**, leading to the identification of peak **11** as astraperocarpan-3-*O*-glycoside (9,10-dimethoxypterocarpan-3-*O*-Glc). The protonated molecular ion at *m/z* 301.1064 and fragmentation pattern of peak **24** coincided with the aglycone ion of peaks **11** and **16**, demonstrating that this compound was 3-hydroxy-9, 10-dimethoxypterocarpan.

Peak **7** generated a [M + H]^+^ ion at *m*/*z* 449.1424 and a [M + NH_4_]^+^ at *m*/*z* 466.1697 corresponding to the molecular formula of C_22_H_24_O_10_. In the MS^2^ data, the typical ions *m*/*z* 287.0924, 259.0976 and 255.0675 generated from [M + H]^+^ by the concurrent loss of C_6_H_10_O_5_, CH_3_OH and CO ion, respectively, indicated peak **7** was a pterocarpan glycoside. After comparing with the literature [26], peak **7** was proposed as licoagroside D (10-dihydroxy-9-methoxypterocarpan-3-*O*-Glc). Peak **20** displayed a precursor ion at *m/z* 287.0911 [M + H]^+^ and the characteristic ions at m/z 259.1, 255.1, 227.1, 177.1, 153.1, 147.0, 138.1 and 123.0 were consistent with the MS^3^ of the aglycone ion of peaks **7**. Thus, peak **20** was tentatively identified as 3, 10-dihydroxy-9-methoxypterocarpan.

Peak **10** gave the precursor ions at *m/z* 535.1428 [M + H]^+^ and *m/z* 552.1690 [M + NH_4_]^+^ (molecular formula of C_25_H_26_O_13_), which were 86 Da (malonyl residue) more than that of licoagroside D (peak **7**), giving the same aglycone ion at *m/z* 287.1. In addition, peak **10** had a similar fragmentation pattern as the astraperocarpan-3-*O*-glycoside-6′-*O*-malonate (**16)** with a decrease of 14 Da (H instead of CH_3_) for the precursor and some fragment ions, confirmed the presence of OH at C-10 in peak **10**. Thus, the structure of peak **10** was presumed to 10-dihydroxy-9-methoxypterocarpan-3-*O*-glycoside-6′-*O*-malonate.

### 2.3. Conversion Analysis of MIGs in Astragali Radix under Different Extract Conditions

The LC-TOF/MS revealed that Astragali Radix contained abundant isoflavonoids, among which MIGs had higher relative peak area. However, a previous study has reported that MIGs were unstable [13]. To clarify the content of MIGs and its possible conversion, six major isoflavonoid components, including two MIGs (calycosin-7-*O*-glycoside-6″-*O*-malonate (CYM) and formononetin-7-*O*-glycoside-6″-*O*-malonate (FMM)) couple with related glycosides (calycosin-7-*O*-glycoside (CYG) and formononetin-7-*O*-glycoside(FMG)) and aglycones (calycosin (CY)and formononetin (FM)), were quantified in different extracts of Astragali Radix using a comprehensive HPLC-PDA method.

#### 2.3.1. Calibration Curves, Linearity, Limits of Detection, and Quantification

Under the optimized chromatographic conditions, all six references calibration curves showed good linear regressions in the range of 1.298–811.4, 0.672–420.0, 0.383–239.1, 0.464–290.1, 0.576–360.0, and 0.470–294.0 μg·mL^−1^, respectively, with the correlation coefficients of 0.9983–0.9995. The analysis of LOD and LOQ showed efficient quantification, which ranged from 0.026–0.047 μg·mL^−1^ and 0.085–0.280 μg·mL^−1^, respectively. The RSD values of inter- and intra-day accuracy of six reference compounds were less than 2.21% and 5.63%, respectively (Table 3). Stability of the Astragali Radix sample was tested at room temperature and analyzed at 0, 2, 4, 6, 8, 10, 12 and 24 h. The results, however, showed that CYM, FMM, CYG and FMG were unstable at room temperature within 24 h; therefore, the references and sample solution should be stored at 4 °C until analysis.

#### 2.3.2. Content Variation of MIGs, Related Glycosides and Aglycones in Different Extraction Samples of Astragali Radix

In this study, two factors, the extraction method and time, which affects the efficiency and stability of the target compounds, were investigated. Extraction methods under different extraction times, including methanol reflux extraction (MFE), water reflux extraction (WFE), and methanol ultrasonic extraction (MUE), were conducted using powdered samples. Then, the effects of the three methods on the concentration of six isoflavonoids were compared. 

**Figure 5:**
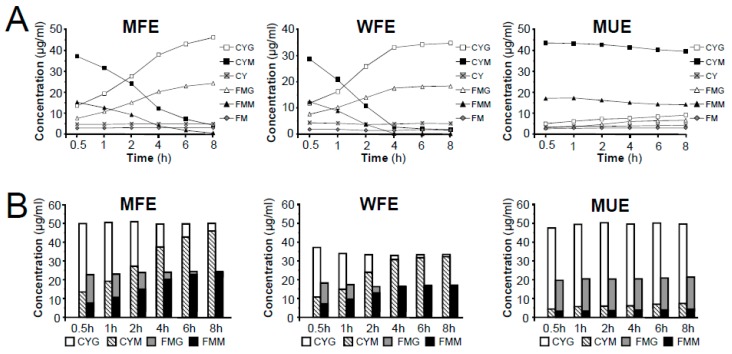
Comparison of extraction efficiency of different methods on the extraction of MIGs, related glycosides and aglycones in Astragali Radix. (**A**) Effects of extraction time on the extraction efficiency of six isoflavonoids in three different methods (MFE, WFE and MUE). (**B**) Effects of extraction method and time on the conversion of the content of matched-constituents (CYG-CYM and FMG–FMM).

By comparing the contents of individual components, the highest yield of the two MIGs (CYM and FMM) was achieved by MUE among all three extraction methods used; conversely, the contents of related glycosides (CYG and FMG) were lower in MUE than in MFE and WFE. Additionally, the contents of CYM and FMM decreased significantly in MFE and WFE during the 8-h extraction, while CYG and FMG showed a remarkable improvement. Furthermore, the contents of two aglycones (CY and FM) in Astragali Radix were relatively low but stable in the three different extraction methods (Figure 5A). In addition, our data showed that the higher the contents of CYM and FMM were, the lower the contents of CYG and FMG observed, which was consistent with the published articles [13]. It suggested that MIGs in raw materials of Astragali Radix might be converted into related glycosides under the elevated temperature conditions, while the glycosides could not be further decomposed into corresponding aglycones. This hypothesis was further confirmed by literatures [12,13] and conversion experiment of reference compounds of CYM and FMM (See Figure S4 and Figure S5 in supplementary materials). The results showed that CYM and FMM only transformed into related glycosides of CYG and FMG, respectively, under methanol reflux extraction condition. Considering the conversion process, although the contents of individual MIGs or related glycosides had a large fluctuation in the procedures of high temperature extraction, the total contents of MIGs and related glycosides should remain basically unchanged. Indeed, we found that the total contents of matched-constituents CYG-CYM and FMG-FMM displayed no obvious change during the long-duration extraction (Figure 5B). In addition, when comparing the total content of CYG-CYM and FMG-FMM, the extraction efficiency of MFE was found to be equivalent to that of MUE and greater than WFE. 

As high content of MIGs were found in this study and the literatures [12,13,14], it is indicated that malonate metabolites are widely spread and highly contained in this crude herbal medicine. However, during the extraction conducted under high temperature conditions, MIGs could transform into corresponding glycosides, resulting in a variation in the content and quality evaluation of Astragali Radix. Fortunately, the total content of MIGs and related glycoside in Astragali Radix remained relatively stable, indicating the quality of Astragali Radix could be evaluated efficiently and accurately by determining the total content of MIGs and related glycosides as the quality index.

## 3. Materials and Methods

### 3.1. Samples, Chemicals and Reagents

Astragali Radix was collected from Baotou County, NeiMenggu Province, China, which is one of the regions in which Astragali Radix is typically cultivated. The samples of Astragali Radix were identified by prof. Xunhong Liuto be the dried roots of *A. membranaceus*. The samples were stored in a dry, dark room atthe School of Pharmacy, Nanjing University of Chinese Medicine.

Reference compounds, including CYG, FMG, CY and FM were purchased from the National Institute for the Control of Pharmaceutical and Biological Products (Beijing, China). Malonyl isoflavonoid glycosides, including FMM, CYM, astraperocarpan-3-*O*-β-D-glycoside-6″-*O*-malonate and astraisoflavanglycoside-6″-*O*-malonate were isolated from the dried roots. The structures were confirmed by comparison of their spectral data (UV, MS,^1^H NMR and ^13^C NMR) with those published in cited references. The purities of the isolated compounds were above 98.0%, as determined by HPLC-PAD using the peak area normalization method.

HPLC grade ACN and CH_3_OH were purchased from Merck (Darmstadt, Germany); distilled water was further purified by Milli-Q system (Millipore, Milford, MA, USA). HPLC grade formic acid was purchased from the Hanbang Company of Nanjing (Nanjing, China).

### 3.2. Isolation and Identification ofMIGs

Astragali Radix (about 5 kg) was exhaustively extracted with methanol percolation (50 L) at room temperature. The extracts were combined and concentrated to 2000 mL under a vacuum at 40 °C. The supernatant was filtered and concentrated to dryness. The residue (a total of about 135 g) was then separated by silica gel CC (1000 g, 200–300 mesh) to three fractions (Fr. A-C) using a gradient elution of EtOAc-MeOH (100:0; 85:15; 70:30, *v*/*v*), respectively. Then, approximately 10.5 g of Fr. C was subjected to middle pressure liquid chromatography (MPLC, BUCHI Chromatography B-688, Flawil, Switzerland) on an RP silica gel column with stepwise solution of MeOH-H_2_O-HCOOH (30:70:0.1–40:60:0.1, *v*/*v*) as the eluent to obtain **AR-1** (103 mg) and **AR-2**(48 mg). Fr. B was then repeatedly separated by MPLC chromatography over an RP-18 silica gel column (400 g, 25–50 μm, 4.5 × 50 cm) eluted with MeOH-H_2_O-HCOOH (30:70:0.1–40:60:0.1, *v*/*v*) to obtain **AR-3** (31 mg) and **AR-4** (26 mg). The purified compounds were characterized by LC-MS and NMR analyses. The MS spectra were recorded on an AB SCIEX Triple TOF^TM^ 5600 mass spectrometer instrument (AB, SCIEX, Los Angeles, CA, USA) in positive ion mode. ^1^H-NMR and ^13^C-NMR spectra were recorded with an ASR-400 NMR spectrometer (Bruker, Fällanden, Switzerland). TMS was used as an internal standard and the specimens were dissolved in DMSO-d_6_ (dimethylsulfoxide).

### 3.3. Chromatographic Methods

Analyses were performed on a Waters Series 2695 liquid chromatography (Waters Technologies, Milford, MA, USA) with an analytical column of Eclipse XDB-C_18_ (4.6 mm × 250 mm, 5 μm, Agilent, Santa Clara, CA, USA). The mobile phase consisted of (A) acetonitrile containing 0.1% (*v*/*v*) formic acid and (B) distilled water containing 0.1% (*v*/*v*) formic acid using a gradient elution: linear from 15 to 32% B (0–25 min), linear from 32 to 62% B (25–50 min). All separations were at 25 °C and a flow-rate of 1.0 mL/min. The injection volume was 5 μL. The HPLC-PDA chromatographic profile was recorded at 260 nm.

### 3.4. LC-TOF/MS Conditions

The mass spectrometry determination was performed on an aquadrupole time of flight mass spectrometer (TripleTOF 5600 system, AB SCIEX) with an electrospray source in the positive ion mode. The automatic data-dependent information product ion spectra (IDA-MS/MS) without any predefinition of the ions were recorded with mass range *m/z* 100–2000. The conditions of ESI source were as follows: nitrogen gas for nebulization at 55 psi, heater gas pressure at 55 psi, curtain gas at 35 psi, temperature of 500 °C, and ion spray voltage at 5500 V in positive ion mode. The acquisition of a survey Q-TOF/MS spectrum was operated under high-resolution settings. The optimized declustering potential (DP) and collision energy (CE) were set at 80 eV and 15 eV in positive ion mode. A sweeping collision energy setting at 35 ± 15 eV was applied for collision-induced dissociation (CID).

### 3.5. Sample Solution Preparation

Methanol ultrasonic extraction (MUE): Approximately 1.0 g dried Astragali Radix powder was weighed and extracted with 20 mL of methanol in an ultrasonic bath for 0.5~8.0 h at room temperature. The supernatant was collected; then stored at a temperature at 4 °C. The extract solution was filtered with 0.45 μm filter membrance before HPLC analysis.

Methanol reflux extraction (MFE): Approximately 1.0 g dried Astragali Radix powder was weighed and extracted with 20 mL of methanol by refluxing in water bath for 0.5~8.0 h at a temperature of 80 °C. After extraction, the glass bottle was kept closed and allowed to cool to room temperature. The supernatant was collected; then stored at a temperature at 4 °C. The extract solution was filtered with 0.45 μm filter membrance before HPLC analysis.

Water reflux extraction (WFE): Approximately 1.0 g dried Astragali Radix powder was weighed and extracted with 20 mL of water by refluxing in water bath for 0.5~8.0 h at a temperature of 100 °C. After extraction, the glass bottle was kept closed and allowed to cool to room temperature. The supernatant was collected; then stored at a temperature at 4 °C. The extract solution was filtered with a 0.45 μm filter membrane before HPLC analysis.

## 4. Conclusions

This was the first systematic investigation of MIGs in Astragali Radix. In this paper, we isolated and purified four MIGs from Astragali Radix, and their fragmentation pathways in ESI/Q-TOF/MS positive mode were proposed. Further research on the chemical profile of MIGs in Astragali Radix led to identify a total of 26 isoflavonoid compounds, including eleven malonyl isoflavonoid glycosides coupled with eight related isoflavonoid glycosides and seven aglycones. This illustrated that the MIGs were an important type of isoflavonoid in Astragali Radix. The quantitative results verified that MIGs could be converted into related glycosides under the elevated temperature conditions, while the total contentdisplayed no obvious change during the long-duration extraction. Our study has provided some technological support for using MIGs and related glycosides to develop quality evaluation methods for Astragali Radix and its products.

## Figures and Tables

**Table 1:** ^13^C-NMR (125 MHz) and ^1^H-NMR (500 MHz) data of compound **AR-1**~**AR-4** in DMSO-d_6_ (**δ** in ppm).

Position	AR-1		AR-2		AR-3		AR-4		Position	AR-1		AR-2		AR-3		AR-4	
	δ_C_	δ_H_ (*J*, Hz)	δ_C_	δ_H_ (*J*, Hz)	δ_C_	δ_H_ (*J*, Hz)	δ_C_	δ_H_ (*J*, Hz)		δ_C_	δ_H_ (*J*, Hz)	δ_C_	δ_H_ (*J*, Hz)	δ_C_	δ_H_ (*J*, Hz)	δ_C_	δ_H_ (*J*, Hz)
2(6)	153.5	8.34(s)	153.7	8.40(s)	65.8	4.28(m); 3.65(m)	69.6	4.19(m); 3.97(m)	5′(10)	119.7	6.97(brs)	113.7	7.00(d,8.0)	133.4	-	103.2	6.46(d,8.5)
3(6a)	123.6	-	123.4	-	39.5	3.65(m)	31.3	3.36(m)	6′(10a)	116.4	7.07(brs)	130.1	7.53(d,8.0)	150.9	-	121.4	6.79(d,8.5)
4(1b)	174.7	-	174.7	-	78.2	5.60(d,6.0)	29.7	2.92(m); 2.81(m)	1″(1′)	99.8	5.13(d,7.5)	99.7	5.15(d,7.5)	100.1	4.86(d,7.5)	100.8	4.79(d,8.0)
5(1)	127.1	8.07(d,8.8)	127.1	8.08(d,8.8)	132.1	7.44(d,8.5)	130.1	7.01(d,8.5)	2″(2′)	76.2	3.34(m)	76.2	3.34 (m)	76.3	3.27(m)	76.3	3.28(m)
6(2)	115.4	7.15(dd,8.8,2.2)	115.4	7.15(dd,8.8,2.2)	110.2	6.72(dd,8.5,2.5)	108.6	6.53(dd,8.5,2.5)	3″(3′)	73.0	3.33(m)	73.0	3.32 (m)	73.1	3.25(m)	73.6	3.23(m)
7(3)	161.2	-	161.2	-	158.3	-	156.4	-	4″(4′)	69.7	3.22(m)	69.7	3.20 (m)	69.8	3.16(m)	70.3	3.17(m)
8(4)	103.6	7.21(d, 2.2)	103.6	7.23(d,2.2)	104.2	6.54(d,2.5)	103.9	6.45(d,2.5)	5″(5′)	73.9	3.76(m)	73.8	3.76(m)	73.7	3.61(m)	74.2	3.59(m)
9(4a)	156.9	-	157.0	-	156.1	-	154.7	-	6″(6′)	64.1	4.41(m); 4.11(m)	64.1	4.41(brs); 4.12(brs)	64.1	4.37(m); 4.08(m)	64.5	4.33(m); 4.10(m)
10(1a)	118.6	-	118.6	-	114.0	-	115.9	-	1‴(1″)	166.9	-	166.9	-	166.8	-	166.2	-
1′(6a)	124.5	-	124.0	-	121.6	-	120.8	-	2‴(2″)	41.5	3.38(s)	41.5	3.40(s)	41.3	3.38(s)	42.3	3.33(s)
2′(7)	112.0	6.97(brs)	130.1	7.53(d,8.0)	118.7	6.99(d,8.0)	148.2	-	3‴(3″)	167.9	-	168.0	-	167.9	-	169.0	-
3′(8)	146.1	-	113.7	7.00(d,8.0)	105.1	6.53(d,8.0)	136.1	-	4′(9)-OCH_3_	55.7	3.80(s)	55.2	3.79(s)	56.0	3.74(s)	56.1	3.75(s)
4′(9)	147.6	-	159.0	-	152.7	-	151.7	-	3′(8)-OCH_3_	-	-	-	-	59.9	3.72(s)	60.9	3.50(s)

The position numbers of **AR-3** are given in brackets. “-”: There is no signals of ^13^C-NMR or ^1^H-NMR.

**Table 2:** MS data for identification of isoflavonoids in *Astragali* *Radix* by (+) HPLC-Q TOF/MS.

Classification	Peak	t_R_ (min)	Molecular Formula	[M + H]^+^/ [M + NH_4_]^+^	[Aglycone + H]^+^	MS^n^ (Characteristic Fragment Ions)	Identification	Reference
Isoflavones	1 *	12.71	C_22_H_22_O_10_	447.1273/-	285.0749 [M + H-Glc]^+^	270.0506, 269.0432, 253.0484, 242.0570, 225.0534, 214.0618, 213.0537, 197.0591, 137.0234	Calycosin-7-*O*-Glc	[12,21]
	2	13.20	C_23_H_24_O_11_	477.1382/-	315.0867 [M + H-Glc]^+^	300.0640, 299.0536, 283.0590, 272.0684, 255.0668, 244.0721, 243.0652, 227.0697, 167.0346	Odoratin-7-*O*-Glc	[21,24]
	4	17.45	C_26_H_26_O_14_	563.1323/-	315.0867 [M + H-Glc-Mal]^+^	300.0635, 299.0571, 283.0631, 272.0675, 255.0650, 244.0731, 243.0654, 227.0695, 167.0328	Odoratin-7-*O*-Glc-6″-*O*-Mal	[21]
	5	17.80	C_25_H_24_O_13_	533.1276/-	285.0750 [M + H-Glc]^+^	270.0523, 269.0451, 253.0500, 242.0570, 225.0546, 214.0624, 213.0548, 197.0597, 137.0243	Isomer calycosin-7-*O*-Glc-6″-*O*-Mal	[21]
	6 *	18.31	C_25_H_24_O_13_	533.1273/-	285.0742 [M + H-Glc-Mal]^+^	270.0506, 269.0434, 253.0477, 242.0567, 225.0530, 214.0615, 213.0534, 197.0588, 137.0229	Calycosin-7-*O*-Glc-6″-*O*-Mal	[12,21]
	8 *	22.77	C_22_H_22_O_9_	431.1327/-	269.0809 [M + H-Glc]^+^	254.0575, 253.0502, 237.0547, 226.0624, 213.0917, 197.0602	Formononetin-7-*O*-Glc (Ononin)	[21,22]
	9	23.05	C_23_H_24_O_10_	461.1426/-	299.0924 [M + H-Glc]^+^	284.0693, 283.0653, 267.0636, 256.0737, 243.1017, 239.0703, 228.0830, 227.0687, 167.0377	6,4′-dimethoxyisoflavone-7-*O*-Glc	[21]
	13	27.84	C_26_H_26_O_13_	547.1421/-	299.0921 [M + H-Glc-Mal]^+^	284.0692, 283.0639, 267.0613, 256.0748, 243.1021, 239.0709, 228.0754, 227.0689, 167.0340	Afrormosin -7-*O*-Glc-6″-*O*-Mal	[24]
	14 *	28.14	C_25_H_24_O_12_	517.1321/-	269.0794 [M + H-Glc-Mal]^+^	254.0564, 253.0486, 237.0538, 226.0620, 213.0904, 197.0595, 137.0232	Formononetin-7-*O*-Glc-6″-*O*-Mal	[12,22]
	15 *	28.87	C_16_H_12_O_5_	285.0749/-	-	270.0517, 269.0446, 253.0495, 242.0580, 225.0547, 214.0623, 213.0542, 197.0600, 137.0240	Calycosin	[12,22]
	22 *	39.50	C_16_H_12_O_4_	269.0803/-	-	254.0582, 253.0499, 237.0551, 226.0631, 213.0918, 197.0603, 137.0251	Formononetin	[12]
	23	40.12	C_17_H_14_O_5_	299.091/-	-	284.0689, 283.0606, 267.0660, 256.0740, 243.1025, 239.0709, 167.0368	Afrormosin (7-hydroxy-6,4′-dimethoxyisoflavon)	[25]
	26	43.20	C_17_H_16_O_4_	285.1117/-	-	270.0527, 269.0439, 242.0590, 214.0621, 213.0546, 153.0202	Biochanin A(5,7-dihydroxy-4′-methoxyisoflavon)	[12]
Isoflavans	3	15.98	C_29_H_38_O_15_	627.2366/ 644.2530	303.1223 [M + H-2Glc]^+^	465.1755, 193.0871, 181.0860, 167.0701, 165.0550, 161.0605, 152.0466, 147.0443, 133.0659, 123.0455	Isomucronulatol-7-*O*-Glc-Glc	-
	12	26.73	C_23_H_28_O_10_	465.1739/ 482.2003	303.1217 [M + H-Glc]^+^	193.0861, 181.0861, 167.0695, 165.0553, 161.0599, 152.0471, 147.0441, 133.0655, 123.0442	Astraisoflavanglycoside (2′-hydroxy-3′,4′-dimethoxy isoflavone-7-*O*-Glc)	[12]
	18 *	30.76	C_26_H_30_O_13_	551.1738/ 568.2004	303.1230 [M + H-Glc-Mal]^+^	515.1529, 497.1435, 411.1429, 193.0858, 181.0858, 167.0692, 165.0545, 161.0600, 152.0473, 147.0545, 133.0655, 123.0441	Astraisoflavanglycoside-6″-*O*-Mal	[12,22]
	19	31.23	C_26_H_30_O_13_	551.1740/ 568.2003	303.1231 [M + H-Glc-Mal]^+^	515.1573, 497.1466, 411.1453, 193.0862, 181.0861, 167.0705, 165.0548, 161.0599, 152.0467, 147.0441, 133.0651, 123.0452	Isomer astraisoflavanglycoside-6″-*O*-Mal	-
	21	34.24	C_25_H_30_O_11_	507.1856/ 524.2102	303.1233 [M + H-Glc-Ac]^+^	471.1678, 453.1582, 411.1440, 193.0845, 181.0864, 167.0706, 165.0552, 161.0602, 152.0471, 147.0444, 133.0661, 123.0453	Isomucronulatol-7-*O*-Glc-6″-*O*-Ac	[21]
	25	41.46	C_17_H_18_O_5_	303.1224/-	-	193.0864, 181.0872, 167.0707, 161.0606, 152.0475, 147.0441, 133.0658, 123.0454	Isomucronulatol	[21]
Pterocarpans	7	18.90	C_22_H_24_O_10_	449.1424/ 466.1697	287.0924 [M + H-Glc]^+^	259.0976, 255.0675, 227.0694, 177.0551, 153.0553, 147.0447, 138.0314, 123.0463	licoagroside D (10-dihydroxy-9- methoxypterocarpan-3-*O*-Glc)	[26]
	10	23.64	C_25_H_26_O_13_	535.1428/ 552.1690	287.0920 [M + H-Glc-Mal]^+^	499.1230, 481.1145, 395.1155, 259.0989, 255.0655, 227.0695, 177.0555, 153.0553, 147.0441, 138.0316, 123.0464	10-dihydroxy-9-methoxypterocarpan-3-*O*-Glc-6′-*O*-Mal	-
	11	25.61	C_23_H_26_O_10_	463.1579/ 480.1841	301.1068 [M + H-Glc]^+^	273.1120, 269.0812, 241.0858, 191.0705, 167.0700, 152.0472, 147.0441, 123.0449	Astraperocarpan-3-*O*-Glc (9,10-dimethoxypterocarpan-3-*O*-Glc)	[12]
	16 *	29.81	C_26_H_28_O_13_	549.1584/ 566.1846	301.1055 [M + H-Glc-Mal]^+^	513.1484, 495.1268, 409.1266, 273.1120, 269.0803, 241.0858, 191.695, 167.0690, 152.0467, 147.0441, 123.0445	Astraperocarpan-3-*O*-Glc-6′-*O*-Mal	[12,22]
	17	30.24	C_26_H_28_O_13_	549.1582/ 566.1847	301.1067 [M + H-Glc-Mal]^+^	513.1564, 495.1265, 409.1257, 273.1117, 269.0809, 241.0868, 191.0704, 167.696, 152.0466, 147.0445, 123.0452	Isomer astraperocarpan-3-*O*-Glc- 6′-*O*-Mal	-
	20	33.58	C_16_H_14_O_5_	287.0911	-	259.0975, 255.0656, 227.0677, 177.0552, 153.0554, 147.0448, 138.321, 123.0456	Vesticarpan (3,10-dihydroxy-9- methoxypterocarpan)	[26]
	24	40.91	C_17_H_16_O_5_	301.1064/-	-	273.1116, 269.0860, 241.0863, 191.0705, 167.0695, 152.0471, 147.0445, 123.0447	3-hydroxy-9,10- dimethoxypterocarpan	[12,24]

Glc = glycoside, Mal = malonate, Ac = acetyl; * these compounds were identified using their corresponding reference standards. “-” there is no signals of MS ions.

**Table 3:** Linear relationships, limit of detection (LOD), limit of quantitation (LOQ) and precision of six isoflavoniod references by HPLC-PDA.

Analyte	Linearity			LOD (ug·mL^−1^)	LOD (ug·mL^−1^)	Precision (RSD, %)	
	Calibration Curve	*R*	Range (ug·mL^−1^)			Inter-Day (n = 3)	Intra-Day (n = 3)
Calycosin-7-*O*-Glc (CYG)	Y = 19248X + 18515	0.9993	1.298~811.4	0.038	0.226	1.96	4.75
Calycosin-7-*O*-Glc-6″-*O*-Mal (CYM)	Y = 13926X + 3619	0.9995	0.672~420.0	0.047	0.280	2.21	5.63
Calycosin (CY)	Y = 29328X + 4448	0.9992	0.383~239.1	0.026	0.085	0.83	2.38
Formononetin-7-*O*-Glc (FMG)	Y = 16433X + 3925	0.9992	0.464~290.1	0.037	0.220	1.88	4.82
Formononetin-7-*O*-Glc-6″-*O*-Mal (FMM)	Y = 14361X + 4344	0.9987	0.576~360.0	0.039	0.235	2.04	5.35
Formononetin (FM)	Y = 26510X + 8997	0.9983	0.470~294.0	0.028	0.092	0.85	2.62

## Supplementary Materials

The following are available online at https://www.mdpi.com/1420-3049/24/21/3929/s1, Figure S1: ESI-Q-TOF/MS (+) spectrum of calycosin-7-*O*-Glc-6″-*O*-Mal (peak 6) and its proposed fragmentations; Figure S2: ESI-Q-TOF/MS (+) spectrum of astraisoflavanglycoside-6″-*O*-Mal (peak 18) and its proposed fragmentations; Figure S3: ESI-Q-TOF/MS (+) spectrum of astraperocarpan-3-*O*-Glc-6′-*O*-Mal (peak 16) and its proposed fragmentations; Figure S4: Conversion analysis of CYM under reflux extraction for 0.5–8.0 h; Figure S5: Conversion analysis of FMM under reflux extraction for 0.5–8.0 h.
