# Supplementary material for: Structural Identification and Conversion Analysis of Malonyl Isoflavonoid Glycosides in Astragali Radix by HPLC Coupled with ESI-Q TOF/MS
Source: Molecules. 2019 Oct 31;24(21):3929. doi: 10.3390/molecules24213929 (PMC6864771; doi:10.3390/molecules24213929)
Supplement: Supplementary file 1 [file molecules-24-03929-s001.pdf]

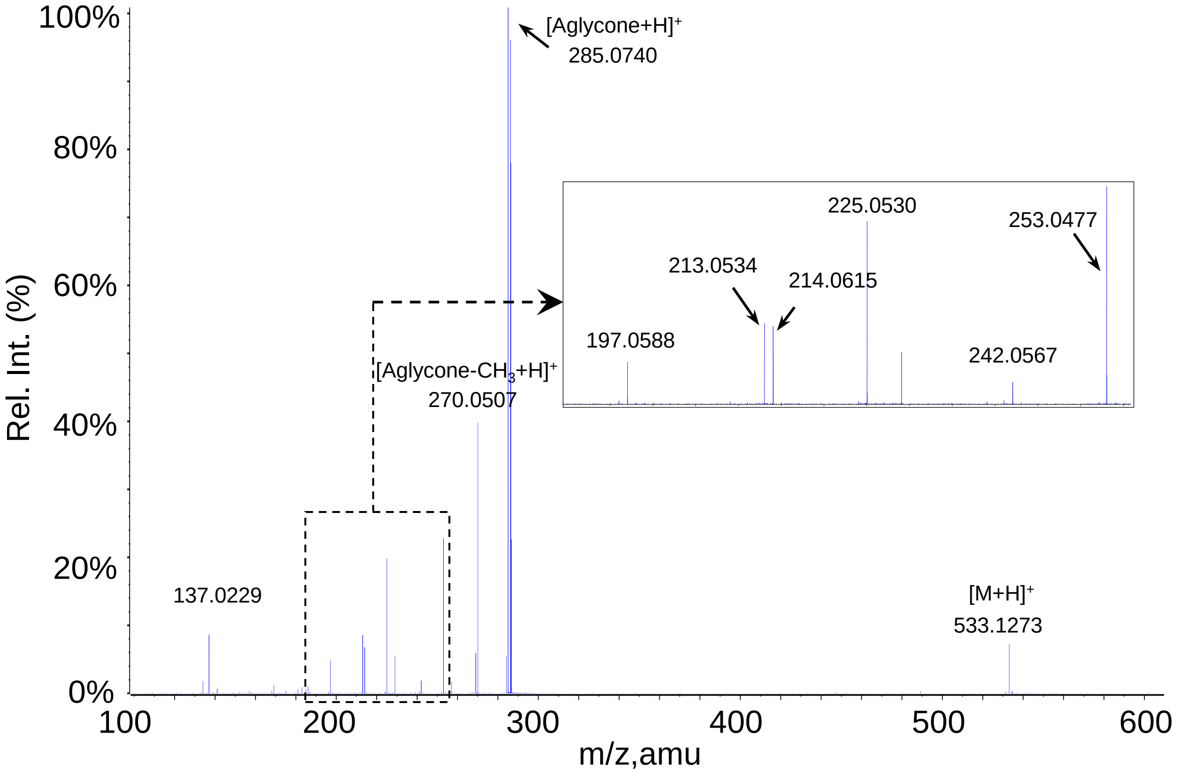

Figure S1. ESI-Q-TOF/MS (+) spectrum of calycosin-7-O-Glc-6″-O-Mal (peak 6) and its proposed fragmentations.

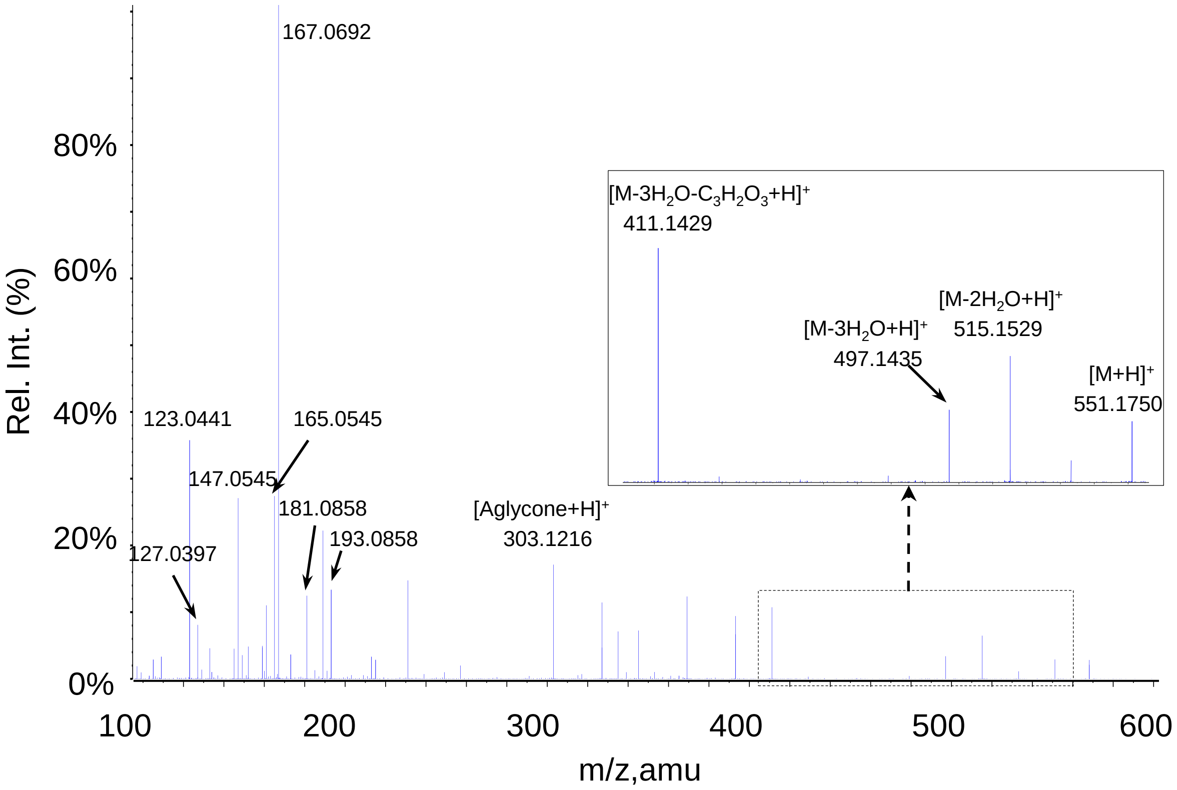

Figure S2. ESI-Q-TOF/MS (+) spectrum of astraisoflavanglycoside-6″-O-Mal (peak 18) and its proposed fragmentations.

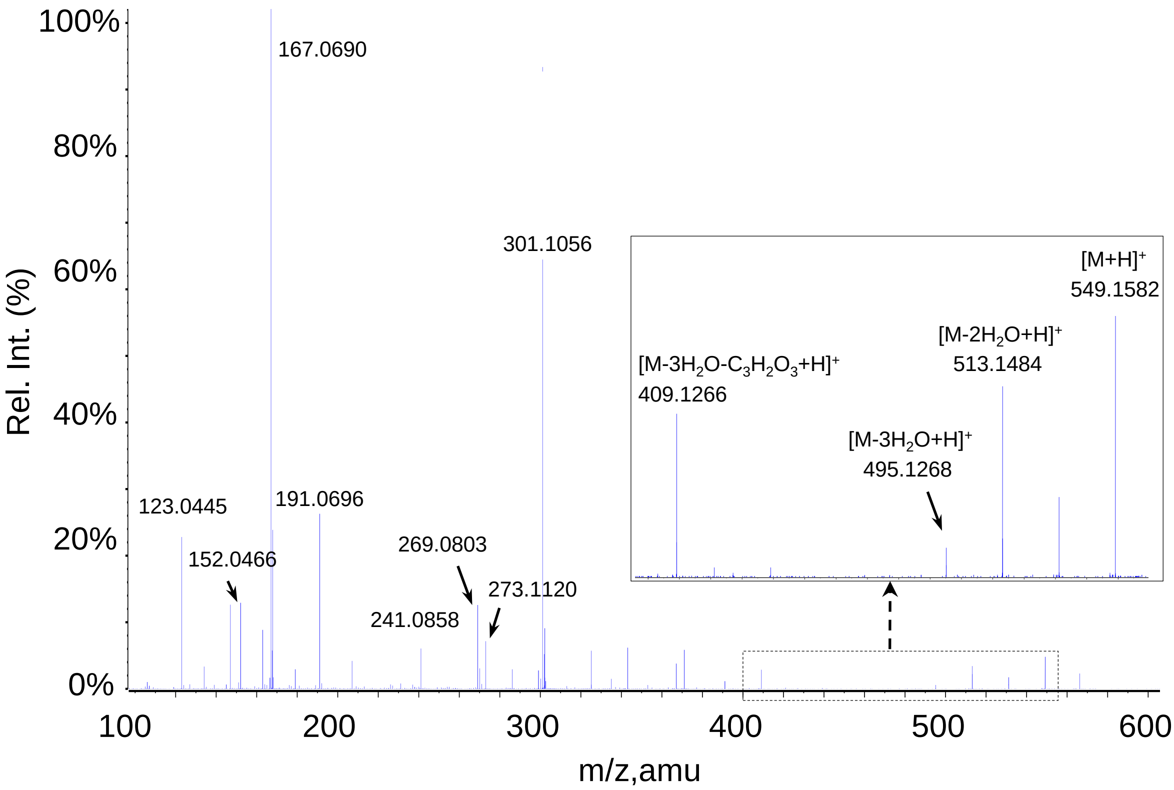

Figure S3. ESI-Q-TOF/MS (+) spectrum of astraperocarpan-3-O-Glc-6′-O-Mal (peak 16) and its proposed fragmentations.

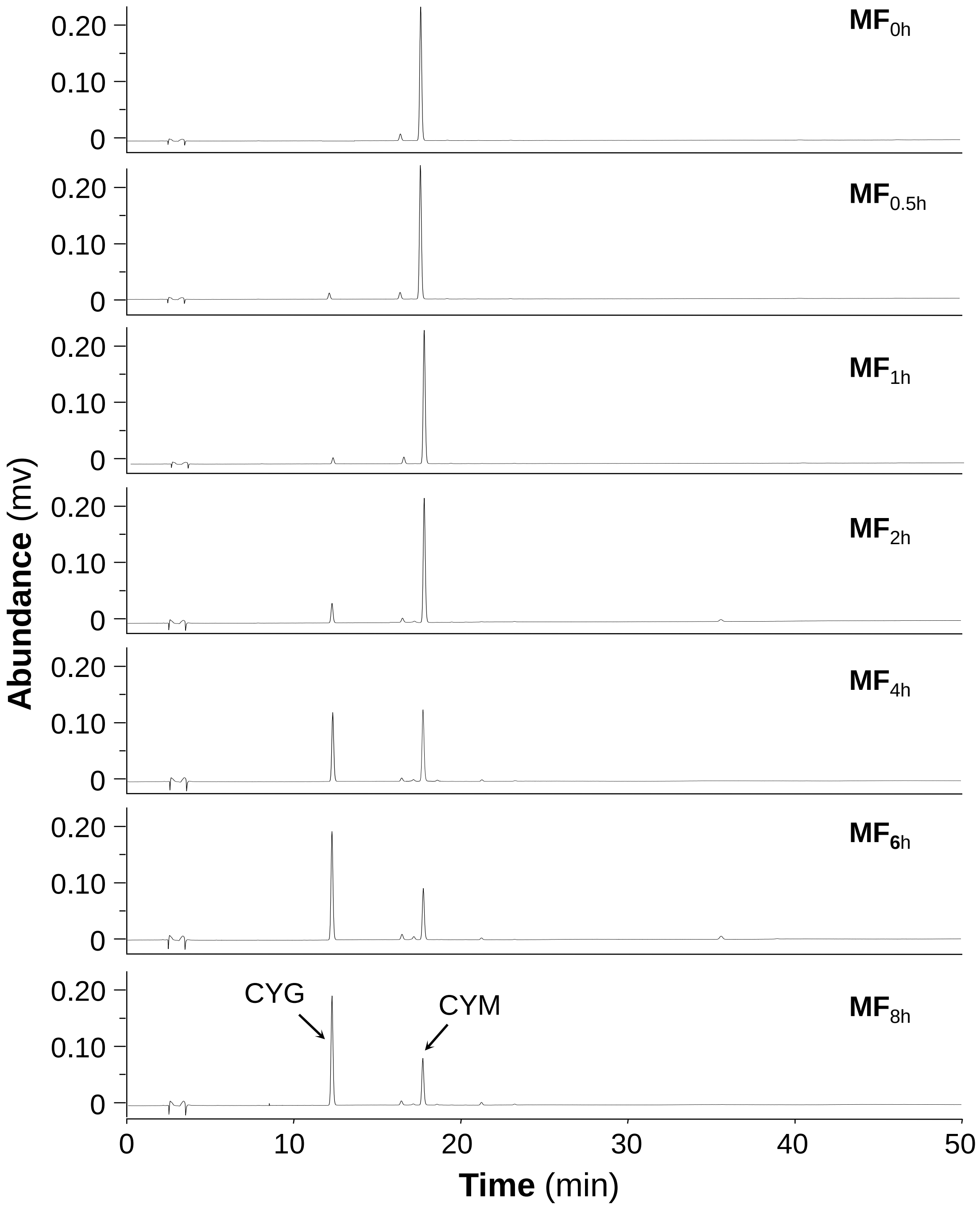

Figure S4. Conversion analysis of CYM under reflux extraction for 0.5-8.0 h

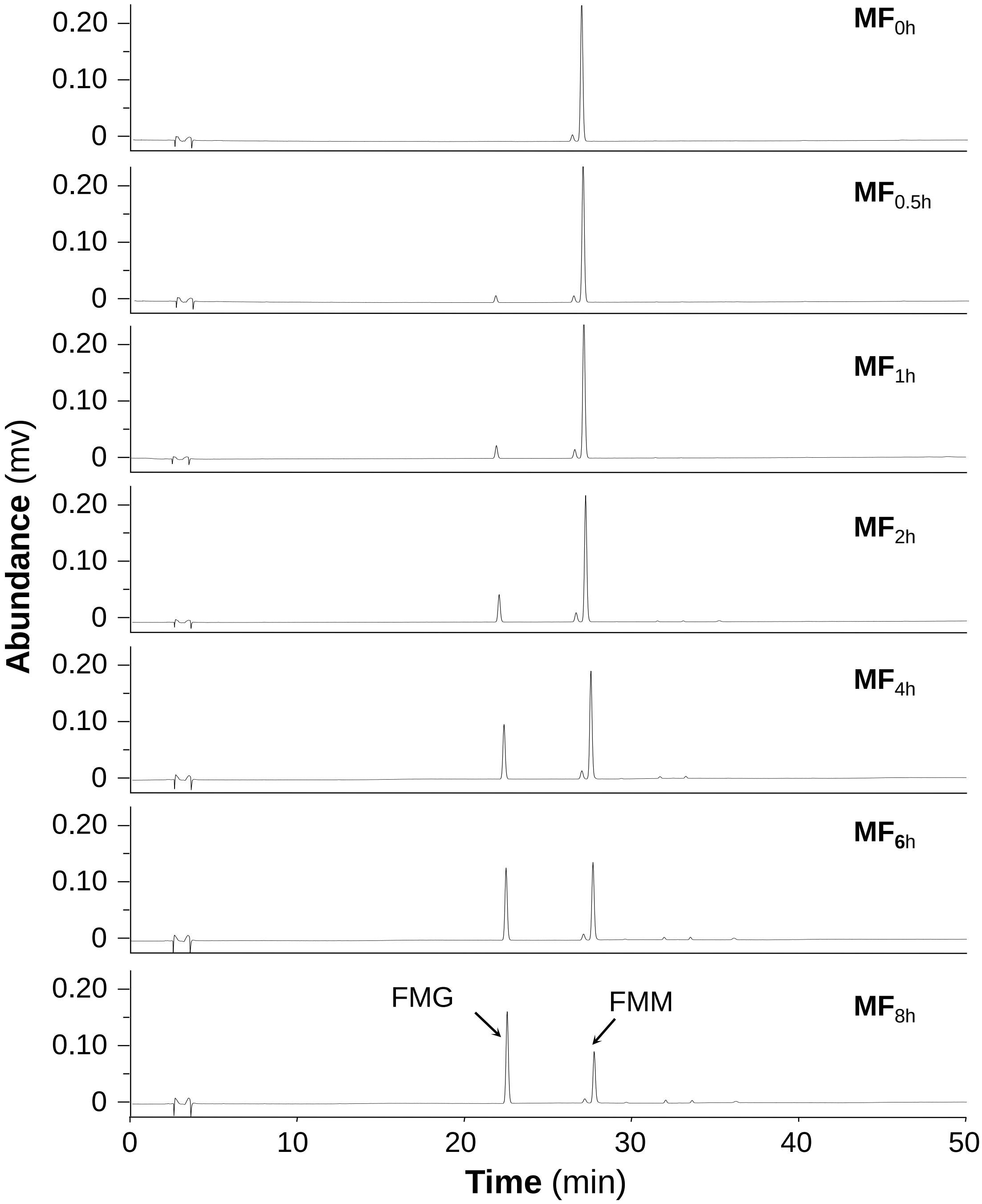

Figure S5. Conversion analysis of FMM under reflux extraction for 0.5-8.0 h
